# The lncRNA TCONS_00021785/miR-21-5p/Trim33 axis regulates VMP1-mediated zymophagy, reduces the activation of trypsinogen, and promotes acinar cell recovery

**DOI:** 10.1038/s41420-022-00862-4

**Published:** 2022-02-15

**Authors:** Qiang Wang, Jingjing Yu, Wenqi Gao, Yuanyuan Sun, Xuxu Liu, Zhenyi Lv, Long Li, Dongbo Xue

**Affiliations:** Department of General Surgery, The First Affiliated Hospital of Harbin Medical University, Harbin, China

**Keywords:** Mechanisms of disease, Necroptosis

## Abstract

In the early stage of acute pancreatitis, trypsinogen in acinar cells is activated, and the cells clear trypsin through zymophagy to avoid damage. Studies have shown that the substrate of zymophagy is ubiquitinated pancreatin, but the mechanism of pancreatin ubiquitination and the regulatory mechanism of zymophagy are not fully understood. Our results show that Trim33 can enhance cell viability, reduce cell necrosis, and reduce trypsinogen activation. Trim33 is a key E3 ligase enzyme that mediates trypsin ubiquitination. The results showed that overexpression of Trim33 can significantly increase VMP1 mRNA and protein levels. However, knocking down Trim33 produced the opposite effect, which indicates that Trim33, as a transcriptional mediator, affects zymophagy by regulating the expression of VMP1. In addition, we explored the transcriptional regulation mechanism of the Trim33 molecule. Our research shows that lncRNA TCONS_00021785 can competitively bind miR-21-5p to upregulate Trim33, thereby initiating enzyme autophagy and reducing zymogen activation.

## Introduction

Acute pancreatitis (AP) is one of the most common gastrointestinal diseases, and its morbidity and mortality are increasing annually [1]. The occurrence of AP involves a variety of complex molecular regulation mechanisms, among which autophagy has always been a concern. At present, it is generally believed that the premature activation of zymogen particles is the main initiating factor of AP. Studies have shown that autophagy is involved in the activation of zymogen particles in acinar cells. Zymogen particles are transported to lysosomes through autophagosomes and accelerate the activation of zymogen particles under the action of acid hydrolase [2, 3]. Mareninova et al. confirmed that it is not the excessive activation of autophagy but the damage of autophagy function that leads to the vacuolization of acinar cells and activation of zymogen [4]. In fact, zymogen activation occurs before autophagy [5]. Enzyme activation to initiate autophagy is the defense mechanism of cells. Effective autophagy can clear the activated zymogen, which plays an important role in maintaining cell homeostasis and the function of pancreatic exocrine secretion [6]. Recent studies have found that in the early stage of acinar cell injury, the activation of pancreatin in the AP model of ElaI-VMP1 mice is significantly reduced [7]; this is a newly discovered type of selective autophagy called zymophagy. However, the molecular mechanism regulating zymophagy is not yet fully understood. For example, what is the enzyme that mediates the ubiquitination of pancreatin? How did zymophagy start? Our research provides some answers to these questions. Revealing the regulatory mechanism of zymophagy is of great significance for preventing the aggravation of AP and developing therapeutic targets for AP.

## Results

### Trim33-mediated ubiquitination plays an important role in rat acinar cells (AR42J cells) treated with TLCS

**Fig. 1:**
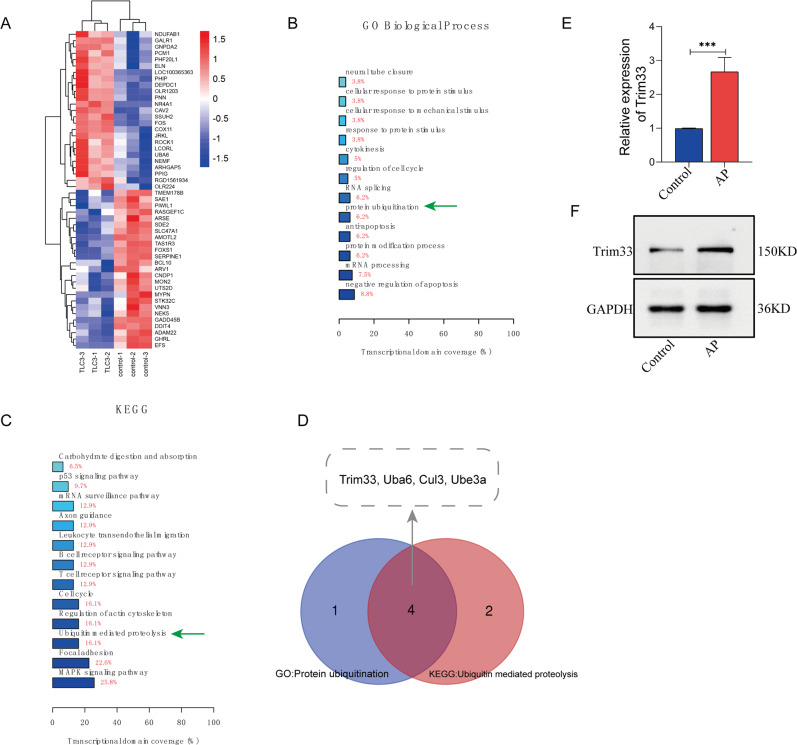
Trim33 was highly expressed in acute pancreatitis and was related to ubiquitin-mediated protein degradation. **A** Gene expression differential analysis (log |FC | > 1, *p* < 0.05); **B** differential gene GO enrichment analysis; **C** differential gene KEGG enrichment analysis; **D** screening of target genes; **E** Trim33 mRNA was highly expressed in AP; **F** Trim33 protein was highly expressed in AP (*t*-test; *** *p* < 0.001).

mRNA biochip detection was performed on AR42J cells treated or not with TLCS. A total of 102 differentially expressed mRNAs were obtained (Fig. 1A). GO/KEGG enrichment analysis was performed on the differentially expressed mRNAs, and significantly enriched GO/KEGG entries were sorted based on the degree of enrichment. We found that “protein ubiquitination” ranked third in the GO biological process category (Fig. 1B) and that “ubiquitin-mediated proteolysis” ranked 3rd in the KEGG pathway category (Fig. 1C). This result indicates that ubiquitination modification is involved in the response of AR42J to TLCS. In the “protein ubiquitination” function, a total of five differential genes were enriched, and a total of six differential genes were enriched in the “ubiquitin-mediated proteolysis” pathway, among which four upregulated differential genes appeared repeatedly (i.e., Trim33, Uba6, Cul3, and Ube3a), and they were identified as candidate genes for further research (Fig. 1D). We then carried out a PPI network of all differential genes (Supplementary Materials, Fig. S1A) and used CytoHub software to perform MCC calculations to screen for hub genes. The results showed that Trim33 was the hub gene among the four ubiquitination-related genes (Supplementary Materials, Fig. 1B). Therefore, we focused on the role of Trim33 in AP. Next, we verified the expression of Trim33 mRNA and protein in the TLCS treatment group (Fig. 1E, F, Fig. S1D).

### Trim33 reversed the decrease in AR42J cell activity caused by TLCS and reduced cell necrosis and trypsinogen activation

**Fig. 2:**
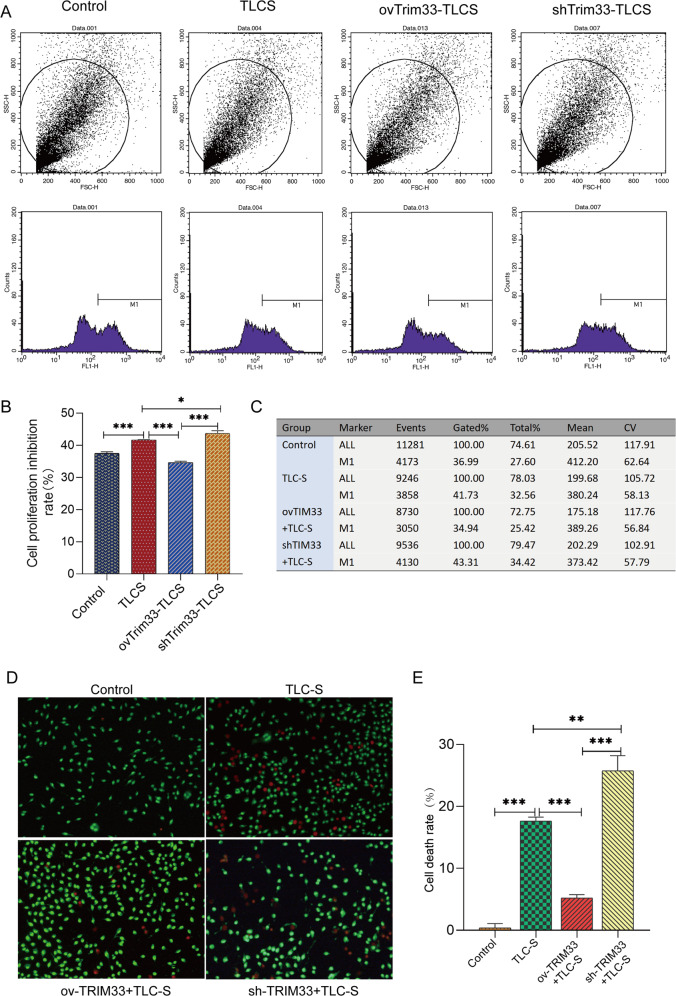
Trim33 can maintain the viability of acute pancreatitis acinar cells and reduce their necrosis. **A**, **B** Cell viability detected by CFSE method, M1 is the cell proliferation inhibition rate. **C** The figure counts the number (Events) and the proportion (Gated%) of the active-deficient cells (M1) in each group. **D**, **E** AO/EB dual fluorescence staining to observe cell necrosis in each group. For the alive cells, the nuclear chromatin are green and show a normal structure; for late apoptosis in cells, the nuclear chromatin are orange-red and pyknotic or fragmented (*t*-test; ***p < 0.001, **p < 0.01, *p < 0.05).

We detected cell viability using the CFSE method, and the results showed that the TLCS treatment group overexpressing Trim33 (ovTrim33-TLCS) had significantly higher cell proliferation activity than the TLCS group (Fig. 2A). In the TLCS treatment group in which Trim33 was knocked down (shTrim33-TLCS), we observed that the cell proliferation inhibition rate was significantly increased. Trim33 reversed the decline in AR42J cell activity caused by TLCS (Fig. 2B). Furthermore, we observed cell necrosis in each group by AO/EB double fluorescent staining. The results showed that compared with the blank control group, the cell necrosis rate in the AP group was significantly increased (Fig. 2D, E). However, in the ovTrim33-TLCS group, the cell necrosis rate was significantly reduced (Fig. 2D, E), and when Trim33 was knocked down, the cell necrosis rate significantly increased (Fig. 2D, E). This indicates that Trim33 can reduce TLCS-induced cell necrosis.

**Fig. 3:**
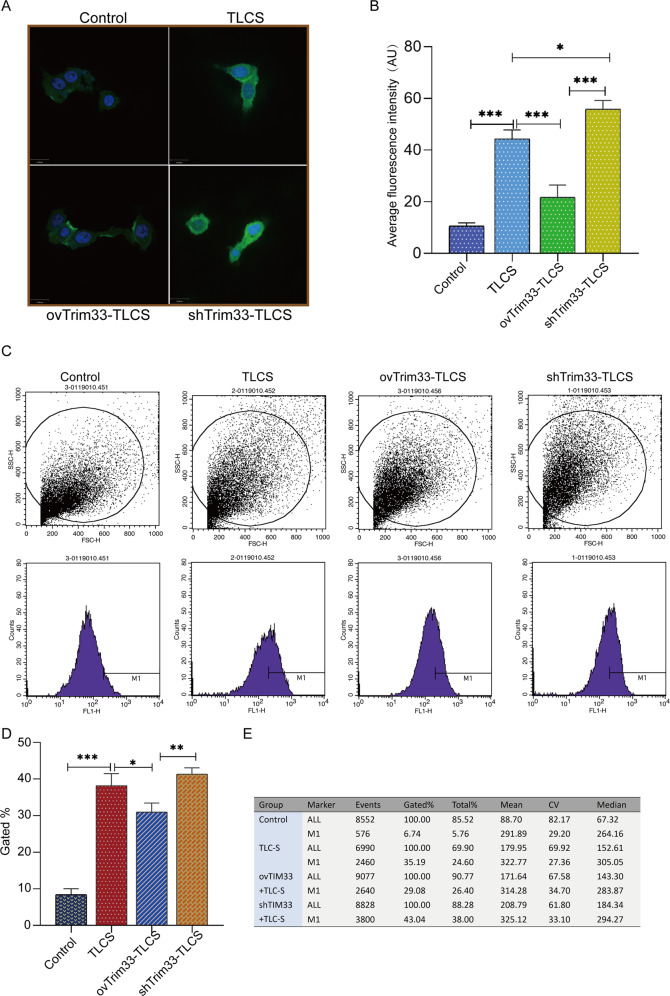
Trim33 can reduce activated trypsinogen. **A** BZiPAR fluorescent substrate method detected the activation of trypsinogen in each group. Green represents BZiPAR, and its fluorescence intensity represents activated trypsinogen, while blue is the cell nucleus (DAPI). **B** The average fluorescence intensity value of each group. **C**, **D** Flow cytometry to detect the fluorescence intensity value of each group. **E** The figure counts the number (Events) and proportion (Gated%) of BZiPAR-stained cells (M1) in each group. (*t*-test; ***p < 0.001, **p < 0.01, *p < 0.05).

We used the BZiPAR fluorescent substrate method to detect the activation of trypsinogen in each group. The average fluorescence intensity reflects the degree of activation of the zymogen. This research suggested that zymogen was significantly activated in the TLCS group, but activated zymogen was significantly reduced in the ovTrim33-TLCS group, and the level of trypsinogen activation was the highest in the shTrim33-TLCS group (Fig. 3A, B). To further verify this result, we tested the activation of each group of zymogens by flow cytometry. The studies suggested that the average fluorescence intensity of the TLCS group significantly increased, indicating that the zymogen was significantly activated; when Trim33 was overexpressed, the fluorescence intensity was significantly reduced, indicating that the activation of the zymogen was reduced; compared with the overexpression group, the fluorescence intensity of the shTrim33-TLCS group significantly increased, which showed that the activated zymogen increased (Fig. 3C–E). This result indicates that Trim33 can reduce the activation of trypsinogen induced by TLCS.

### Trim33 mediates trypsin ubiquitination to initiate the zymophagy process of trypsin

**Fig. 4:**
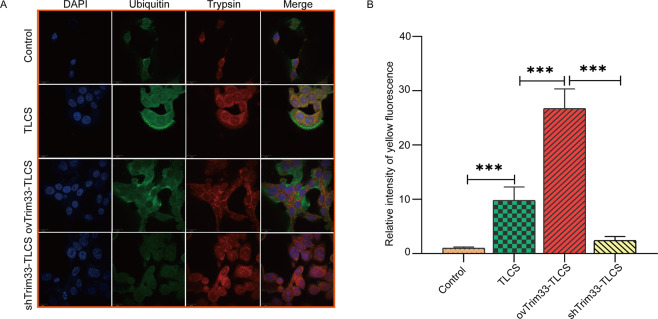
Trim33 can promote the ubiquitination of activated zymogen. **A** Co-localization analysis of endogenous ubiquitin and zymogen particles, showing DAPI-labeled nucleus, green fluorescein-labeled ubiquitin antibody, red fluorescein-labeled trypsin, and yellow fluorescence represents the co-localization of ubiquitin and activated zymogen; **B** the relative intensity of yellow fluorescence in each group (*t*-test, ***p < 0.001).

**Fig. 5:**
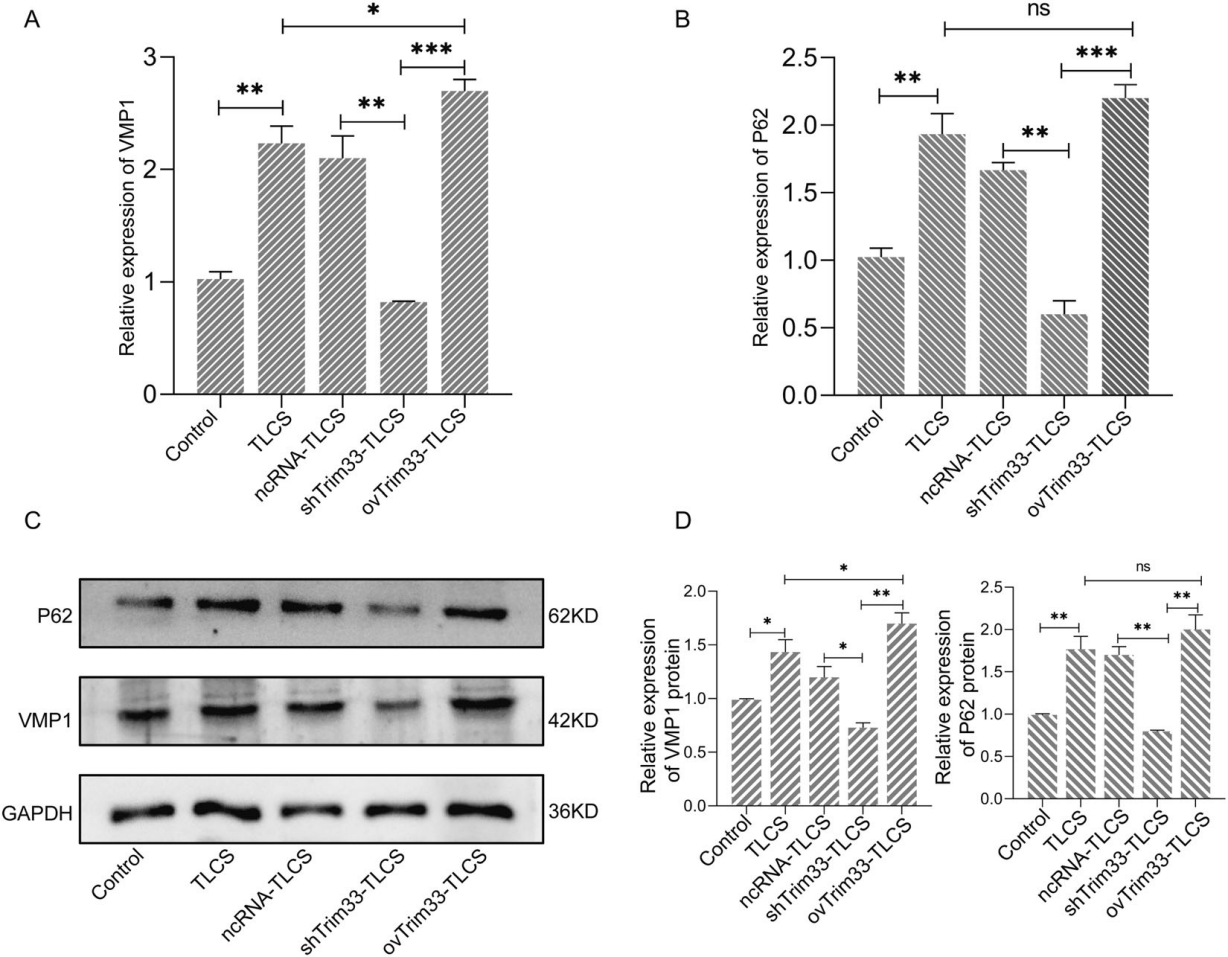
Trim33 can activate zymophagy and regulate its activity and efficiency. **A** The mRNA expression levels of VMP1; **B** the mRNA expression levels of P62; **C**, **D** the protein levels of VMP1 and P62 (*t*-test, ***p < 0.001, **p < 0.01, *p < 0.05).

We hypothesize that Trim33 mediates trypsin ubiquitination to initiate the zymophagy process of trypsin. To verify this hypothesis, we performed ubiquitination analysis on the activated zymogen (Fig. 4A). The semiquantitative results showed that the activated zymogen particles in the ovTRIM33-TLCS group were significantly ubiquitinated (Fig. 4B), and the trypsinogen activity was reduced. When Trim33 was knocked down, trypsin ubiquitination was significantly reduced, and zymogen activation increased. This result indicates that Trim33 is a key molecule that promotes the ubiquitination of activated zymogen particles in pancreatic acinar cells. We overexpressed or knocked down Trim33 in AR42J cells and then detected the mRNA and protein levels of VMP1 and P62 to evaluate whether zymophagy was triggered and the level of activity and the efficiency of zymophagy. The autophagosome-associated protein VMP1 is a transmembrane protein that is not expressed in cells where zymophagy is not activated; zymophagy activation can induce cells to express VMP1. The expression level of VMP1 can reflect the number of autophagosomes. P62 protein is specifically degraded by the autophagy pathway, so the analysis of P62 protein expression changes can reflect the activity and efficiency of autophagy. The results showed that compared with the TLCS group, the levels of VMP1 mRNA in the ovTirm33-TLCS group were significantly increased (Fig. 5A), but there was no significant difference in the expression of P62 between the two groups (Fig. 5B). The WB results showed that VMP1 was increased significantly in the ovTirm33-TLCS group, but the level of P62 protein did not increase significantly (Fig. 5C, Fig. S1E). However, in the shTrim33-TLCS group, the mRNA and protein levels of VMP1 and P62 were significantly lower than those in the TLCS group and ovTirm33-TLCS group (Fig. 5A–D). This indicates that Trim33 is essential for the expression of VMP1 and P62 and that the triggering of zymophagy depends on Trim33.

### The miR-21-5p/Trim33 axis can inhibit zymophagy by increasing activated zymogen

**Fig. 6:**
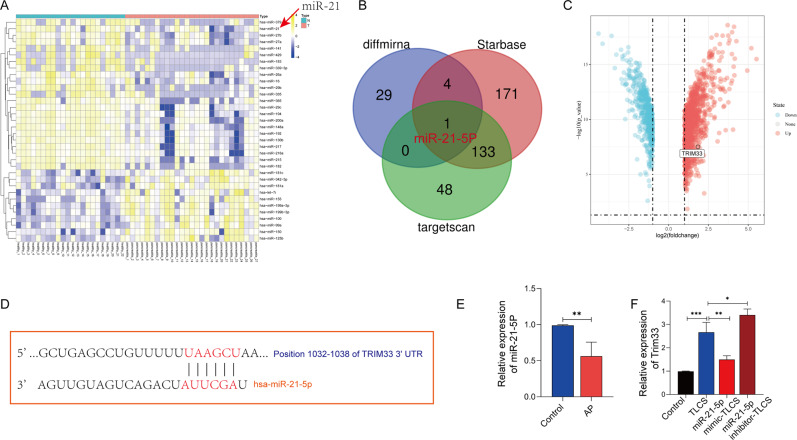
miR-21-5p can inhibit the expression of Trim33. **A** miRNA difference analysis; **B** starBase and TargetScan predicted that miRNA may bind to Trim33; **C** analyzing the mRNA difference between human AP tissue and normal tissue; **D** the binding site of miR-21-5p and Trim33; **E** the expression of miR-21-5p in the AP group was significantly lower than that in the control group; **F** miR-21-5p was overexpressed and knocked down in AR42J cells and treated with TLCS, and then the mRNA and protein levels of Trim33 were observed (*t*-test, ***p < 0.001, **p < 0.01, *p < 0.05).

**Fig. 7:**
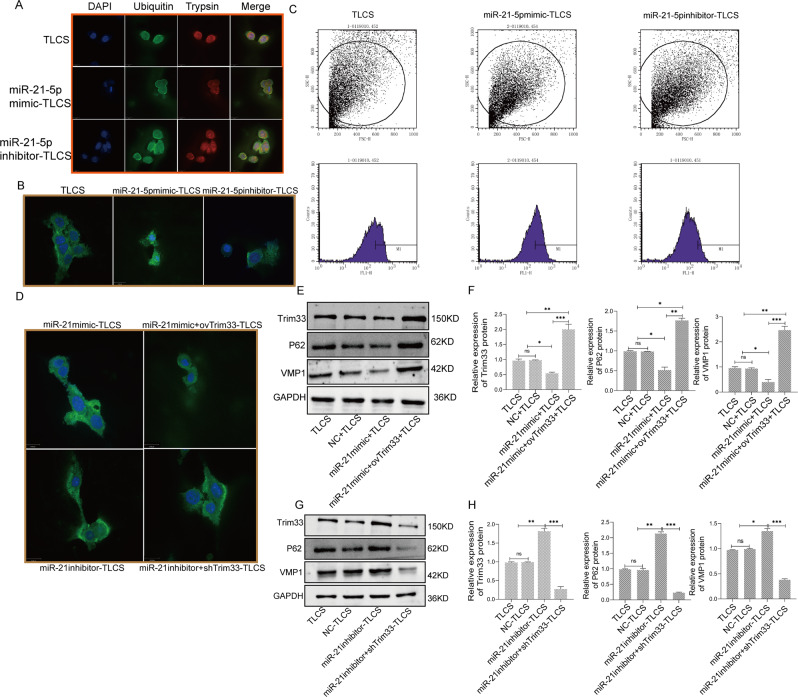
miR-21-5p inhibits zymophagy by regulating Trim33. **A** Co-localization analysis of endogenous ubiquitin and zymogen particles. **B** The photos of the activation of each group of zymogen particles were taken by a confocal microscope. **C** Flow cytometry to detect the fluorescence intensity value of each group. **D** Detect the activation level of zymogen in each group. **E**–**H** The mRNA and protein levels of VMP1,Trim33 and P62 in each group were detected by RT-PCR and Western blot (*t*-test, ***p < 0.001, **p < 0.01, *p < 0.05).

We obtained miRNA sequencing data (GSE24279) from normal individuals and AP patients from the GEO database. We performed a difference analysis between the normal group and the AP group, and we found that there were 34 different miRNAs, of which 11 were upregulated and 23 were downregulated (Fig. 6A). The miR-21-5p was obtained by intersecting the miRNAs of the two databases (Fig. 6B). To maintain the homology of the species, we analyzed the mRNA differences between human AP tissues and normal tissues. We found that the expression of Trim33 was also increased in human AP tissues (Fig. 6C), which is consistent with our sequencing results (Fig. 1A). Therefore, we believe that miR-21-5p may have a regulatory effect on Trim33. Fig 6D shows the binding sites of miR-21-5p and Trim33. To verify the regulatory effect of miR-21-5p on Trim33, we overexpressed or knocked down miR-21-5p in AR42J cells, treated them with TLCS, and then observed the mRNA and protein levels of Trim33, the level of trypsin ubiquitination, and the level of pancreatin activation. Compared with the AP group, overexpression of miR-21-5p significantly inhibited the mRNA (Fig. 6F) and protein expression (Fig. 7E, F) of Trim33, reduced the level of trypsin ubiquitination (Fig. 7A), and reduced the level of activated trypsinogen (Fig. 7B, C). Knockdown of miR-21-5p indicated the opposite trend. We conducted rescue experiments. Compared with the overexpression miR-21-5p group, the restoration of Trim33 on this basis increased the ubiquitination of trypsin and reduced the activated zymogen (Fig. 7D). Similarly, knocking down miR-21-5p and silencing Trim33 reduced trypsin ubiquitination and increased activated zymogen (Fig. 7D). Next, we verified that miR-21-5p regulates zymophagy by inhibiting Trim33. We tested the mRNA and protein levels of VMP1 and P62 in each group. The studies suggested that overexpression of miR-21-5p inhibited the expression of VMP1 and P62 (Fig. 7E, F, Fig. S1F). Knockdown of miR-21-5p increased the expression of VMP1 and P62 (Fig. 7G, H, Fig. S1G).

### lncRNA TCONS_00021785 can competitively bind miR-21-5p to upregulate Trim33

**Fig. 8:**
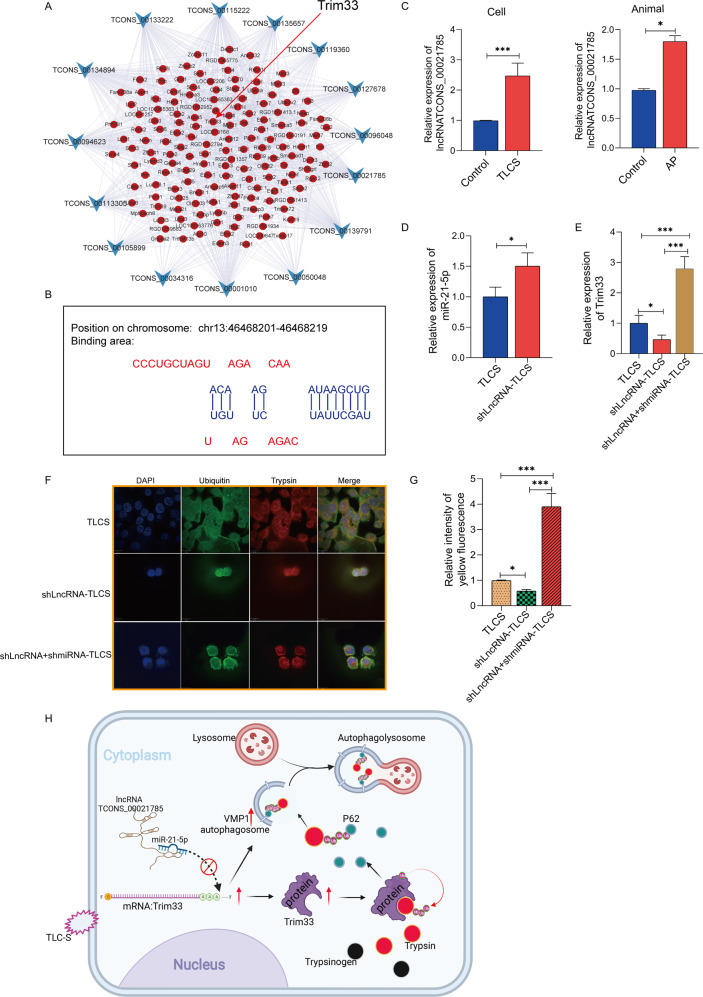
lncRNA TCONS_00021785 can bind miR-21-5p to regulate Trim33. **A** Construction of a co-expression network of LncRNA and mRNA; **B** predicted lncRNA TCONS_00021785 can bind miR-21-5p; **C** RT-PCR confirmed that lncRNA TCONS_00021785 was highly expressed in the AP cell model and the AP rat model; **D**, **E** detect the expression of miR-21-5p and Trim33; **F** detect the activation of trypsinogen in each group. **G** Its fluorescence intensity represents the activated trypsinogen. **H** When acinar cells are damaged by external stimuli, lncRNA TCONS_00021785 can competitively bind miR-21-5p to upregulate Trim33 (*t*-test, ***p < 0.001, **p < 0.01, *p < 0.05).

We proved that miR-21-5p could inhibit the expression of Trim33, but in fact, Trim33 is highly expressed in AP. Therefore, we believe that there are molecules in AP that compete with Trim33 to bind to miR-21-5p. In recent years, a large number of studies have reported that long noncoding RNAs can compete with mRNAs to bind to miRNAs through the ceRNA mechanism [8]. Based on the microarray data of lncRNAs and mRNAs, we constructed a co-expression network for the upregulated lncRNAs and mRNAs (Fig. 8A). We found that Trim33 expression was positively correlated with the expression levels of 11 lncRNAs.Which of these 11 potential lncRNAs have miR-21-5p binding sites? We made predictions through bioinformatics and found that lncRNA TCONS_00021785 can bind to miR-21-5p (Fig. 8B). First, we confirmed that lncRNA TCONS_00021785 was indeed highly expressed in the AP cell model and the AP rat model (Fig. 8C). To confirm the regulatory relationship of lncRNA TCONS_00021785/miR-21-5p/Trim33, we silenced lncRNA TCONS_00021785 in vitro and detected the expression of miR-21-5p and Trim33. The results showed that after lncRNA TCONS_00021785 was silenced, miR-21-5p expression was significantly increased, and Trim33 had significantly reduced expression (Fig. 8D). To show that lncRNA TCONS_00021785 could bind to miR-21-5p to regulate Trim33, we silenced miR-21-5p by silencing lncRNA TCONS_00021785 and observed the expression of Trim33. The results showed that Trim33 was significantly upregulated (Fig. 8E). This fully proved the regulatory relationship among the three. We further tested the effect of lncRNA TCONS_00021785/miR-21-5p on Trim33 function. In the lncRNA TCONS_00021785 silencing group and the lncRNA TCONS_00021785–miR-21-5p simultaneous silencing group, we observed the ubiquitination level of the activated zymogen and the activation degree of the zymogen. We found that in the lncRNA TCONS_00021785-silenced group, compared with the control group, the ubiquitination level of activated zymogen was significantly reduced (Fig. 8F, G), and the degree of zymogen activation was significantly increased (Fig. 8F, G). However, the knockdown of miR-21-5p rescued the ubiquitination of the activated zymogen and, thus, reduced zymogen activation (Fig. 8F, G).

## Discussion

Trim33 is a member of the TRIM family and it interacts with the Ub-coupled enzyme (E2) through its RING domain and transfers Ub from the inactivated enzyme (E1) to the target molecule [9, 10]. Studies have shown that Trim33 binds to and induces DHX33 ubiquitination to promote the formation and activation of the DHX33–NLRP3 complex [11]. In addition, Trim33 has been shown to play a role in transcriptional regulation during hematopoiesis [12]. According to previous reports, it can inhibit the metastasis of hepatocellular carcinoma and exert tumor suppressor activity [13]. Recent studies have shown that Trim33 is related to DNA damage repair [14].

Prior to this, the role of Trim33 in acute pancreatitis was unclear. Our research found that Trim33 is the hub gene of AP, being highly expressed in AP (Fig. 1). Functional analysis revealed that Trim33 is involved in the degradation of ubiquitinated proteins in pancreatitis (Fig. 1). First, we confirmed that Trim33 has a protective function in acinar cells stimulated by TLCS. Our results indicate that overexpression of Trim33 can enhance AR42J cell viability (Fig. 2B), reduce cell necrosis (Fig. 2D) and trypsinogen activation (Fig. 3). Trim33 is a RING-type E3 ubiquitin ligase that can connect activated ubiquitin to its substrate [15]. Additional results showed that Trim33-mediated trypsin ubiquitination is a key step in activating trypsin reduction in cells (Fig. 4).

Zymophagy can reduce ubiquitinated trypsin6. Does Trim33 act as E3 to participate in the zymophagy process? We explored this issue. The degradation of intracellular proteins occurs mainly through two pathways, namely, the autophagy–lysosome system and the ubiquitin–proteasome system (UPS) [16, 17]. However, the regulatory relationship between the UPS and autophagy has not yet been fully clarified [18]. Studies have shown that in a mouse model of acute pancreatitis, after pancreatin is ubiquitinated, p62 acts as a cargo receptor to link ubiquitinated trypsin to the vmp1-autophagosome to initiate zymophagy [6]. However, there has been no previous report that trypsin is ubiquitinated by that molecule. There is no evidence that vmp1 in autophagosomes is regulated by this molecule. Our research results confirmed that Trim33, as an E3 ligase, can ubiquitinate activated zymogens. Trim33, as a transcriptional mediator, can regulate the expression of VMP1. These results showed that overexpression of Trim33 significantly increased VMP1 mRNA and protein levels (Fig. 5A, B). However, knocking down Trim33 produced the opposite result (Fig. 5C). In conclusion, we confirmed that Trim33 ubiquitinates trypsin and initiates zymophagy.

We confirmed that increasing the expression of Trim33 can enhance the activity and efficiency of zymophagy. The expression of Trim33 is increased during acute pancreatitis, but its molecular expression regulation mechanism is still unclear. It is well-known that miRNAs can regulate protein-coding genes by targeting the sequence of the 3’-UTR of the target gene and affect the translation and expression of the protein [19–21]. The regulatory mechanism of miRNA on autophagy has been reported in many studies. The literature has shown that miR-376b44, miR-216a [22], and miR-30b [23] inhibit the expression of beclin1 and thus impede the initial formation of autophagosomes. MiR-204 inhibits autophagosome extension by directly targeting LC3 [24]. We found through bioinformatics that miR-21-5p regulated the expression of Trim33 (Fig. 6A–D). RT-PCR confirmed that miR-21-5p was highly expressed in AP (Fig. 6E). To verify the regulatory effect of miR-21-5p on Trim33, we overexpressed or knocked down miR-21-5p in AR42J cells and then observed the mRNA and protein levels of Trim33. Our results confirm that Trim33 is regulated by miR-21-5p (Fig. 6F). In addition, we verified this conclusion through rescue experiments (Fig. 7).

In recent years, a large number of studies have reported that long noncoding RNAs can compete with mRNAs to bind miRNAs through a ceRNA mechanism [8]. We found that lncRNA TCONS_00021785 could bind miR-21-5p through the coexpression network and site prediction (Fig. 8A, B). First, we confirmed that lncRNA TCONS_00021785 was indeed highly expressed in the AP cell model and the AP rat model (Fig. 8C). After silencing lncRNA TCONS_00021785, miR-21-5p expression was significantly increased (Fig. 8D), while Trim33 expression was significantly reduced (Fig. 8E). Further rescue experiments confirmed that lncRNA TCONS_00021785 can bind miR-21-5p to regulate Trim33 (Fig. 8E). In addition, we verified that the lncRNA TCONS_00021785/miR-21-5p/Trim33 axis regulates the level of trypsinogen activation and pancreatin ubiquitination (Fig. 8F, G).

## Conclusion

This study showed that when AP occurs, the body activates zymophagy to repair the damaged pancreas. This tends to promote AP self-healing, avoiding deterioration. When acinar cells are damaged by external stimuli, lncRNA TCONS_00021785 can competitively bind miR-21-5p to upregulate Trim33. Trim33, as an E3 ligase, can ubiquitinate activated zymogens to initiate zymophagy. Trim33, as a transcription mediator, can regulate the expression of VMP1 and zymophagy (Fig. 8H).

## Materials and methods

### Cell culture and mRNA microarrays

The ar42J cell line was obtained from The Cell Bank of Type Culture Collection of the Chinese Academy of Sciences. Cells were cultured in F-12k (Gibco; Thermo Fisher Scientific, Inc.) and supplemented with 10% FBS (Gibco; Thermo Fisher Scientific, Inc.), 100 µg/ml streptomycin, and 100 µ/ml penicillin (Beyotime Institute of Biotechnology), and then placed at 37 °C in a humidified incubator containing 5% CO_2_. The cells in the TLCS group were treated with 200 µM TLCS (Sigma-Aldrich; Merck KGaA) for 40 min at 37 °C [21, 22], whereas cells in the control group were left untreated. Total RNA was extracted from cells using Trizol^®^ reagent (Invitrogen; Thermo Fisher Scientific, Inc.). Gene expression analyses were performed via Rat Microarray v2.0 (cat. no. Agilent-062716; Agilent Technologies, Inc.).

### Animals

The Animal Research Center of the First Clinical College of Harbin Medical University provided 10 healthy male SD rats (250 ± 20 g). The rats were randomly divided into two groups and allowed to acclimate for one week. The rats fasted with free access to water for 12 h before surgery. A midline incision was made along the linea alba, and the pancreatic duct was exposed at the junction between the stomach and the duodenum. Acute pancreatitis was induced by a retrograde infusion of 3.5% sodium taurocholate (Na-TC, 0.15 ml/100 g) into the pancreaticobiliary duct. In the control group, ligation of the pancreatic duct was not performed, and an equal volume of sterile saline was injected intraperitoneally.

### Reverse transcription-polymerase chain reaction (RT-PCR)

SYBR GreenIReal Time PCR was used to detect the expression levels of Trim33, miR-21-5p, and lncRNA TCONS_00021785 in rat pancreatic acinar cells. For the real-time PCR detection of target gene primers, the following primers were synthesized by the Beijing Invitrogen Company: lncRNA TCONS_00021785 upstream primer GGCATCTTGAATCTTGCTTTG; lncRNA TCONS_00021785 downstream primer CAACATTTCAGGACGAGACACT; Trim33 upstream primer CGTCTTCTGCTTTACCTCTATTGC; Trim33 downstream primer GGACATCAGCCACAAAGTCATCT; GAPDH upstream primer TTCCTACCCCCAATGTATCCG; GAPDH downstream primer CCACCCTGTTGCTGTAGCCATA; Wbrno-miR-21-5p upstream primer TAGCTTATCAGACTGATGTTGAAAA; rno-miR-21-5p downstream primer GTGCGTGTCGTGGAGTCG; U6 upstream primer CTCGCTTCGGCAGCACA; U6 downstream primer AACGCTTCACGAATTTGCGT.

### Western blotting

AR42J cells were lysed using RIPA buffer (cat. no. P0013K; Beyotime Institute of Biotechnology), and the protein concentration was measured using a bicinchoninic acid protein assay kit (cat. no. P0011; Beyotime Institute of Biotechnology). Proteins (40 µg) were separated via 8% sodium dodecyl sulfate-polyacrylamide gel electrophoresis and transferred onto 0.45 µm polyvinylidene fluoride membranes. Membranes were blocked with 5% skimmed milk dissolved in PBST for 1 h at room temperature. Membranes were incubated with SQSTM1 monoclonal antibody (Product #MA5-31498, 1:500) and VMP1 polyclonal antibody (Product #PA5-77784, 1:1000) at 4 °C overnight. Membranes were then incubated with horseradish peroxidase secondary antibody (1:2000; cat. no. ZB-2301; ZSGB-Bio; oriGene Technologies, Inc.) for 1 h at room temperature. An ECL kit (cat. no. P0018; Beyotime Biotech, Inc.) was used to detect the signal on the membrane. Data were analyzed by densitometry using Image Lab software (v.2.0.1; Bio-Rad Laboratories, Inc.) and normalized to the expression of the internal control GAPDH.

### Cell transfection

AR42J cells were planted in a 6-well plate, and after 8–12 h, the cell growth density and cell growth state were observed under a microscope. When the cells were in good condition and the growth density was approximately 60–80%, transfection was ready to begin. The mir-21-5p mimics, inhibitor, TRIM33si-RNA, plasmid, Lip2000, and Opti-MEM were prepared in advance on an icebox and then put into a super-clean table together with other essential items (such as a transfer gun, sterilized gun head, and sterile EP tube). At the same time, the super-clean table was wiped with cotton balls sterilized in alcohol, and the UV lamp was turned on for 30 min and then turned off for 10 min for ventilation. Two sterile EP tubes were readied to prepare the transfection reagents. Four microliters of si-RNA were added to 100 μl of Opti-MEM; 4 μl of Lip2000 and 100 μl Opti-MEM were added to the other tube, and the mixtures were placed for at least 5 min at room temperature. The contents of the two tubes were mixed until even, namely, to 200 μl, and both were placed at room temperature for 15–20 min. The culture medium was removed, washed once with phosphate-buffered saline (PBS), and 800 μl of new complete medium was added. After adding the transfection mixture of 200 μl and shaking well, the cells were returned to the incubator and cultured for 24 h, and the samples were collected to extract RNA and protein.

### Laser confocal microscopy to evaluate trypsinogen activation

The cells in the confocal dish were washed with PBS 2–3 times; 150 μL of 4% paraformaldehyde was added for 15 min at room temperature to fix the cells. After being washed with PBS 2–3 times, the cells were incubated with PBS containing 2% Tween-20 for 10 min at room temperature. After being washed with PBS again 2–3 times, BZiPAR staining solution was added at room temperature in the dark for 60 min. After being washed with PBS another 2–3 times, the cells were directly observed under a laser confocal microscope (A1R, Nikon, Japan).

### Immunofluorescence co-localization to assess the ubiquitination of activated zymogen

AR42J cells were fixed with paraformaldehyde, permeabilized with 0.1% Triton X-100, blocked with 10% serum (45 min at 25 °C), incubated with mouse alpha-1 Antitrypsin Antibody (PA1-22860) and Ubiquitin polyclonal antibody (PA5-102555) using a dilution of 1:200 (1 h, 37 °C), and followed by goat anti-rabbit IgG Alexa Fluor 594 (red) and goat anti-rabbit IgG Alexa Fluor 488 (green).

### Flow cytometry to detect the activation of trypsinogen

After the cells were digested and collected, 1 × 10^6^ cells from each group were centrifuged at 1000 rpm for 5 min. The supernatant was discarded before 500 μL of 1× PBS was added to wash the sample two times, with centrifugation at 1000 rpm for 5 min for each wash. The cells were then resuspended with 200 μL of BZiPAR (CBZ-Ile-Pro-Arg) 2-rhodamine 110 (Molecular Probes, USA) working solution, followed by reaction at room temperature in the dark for 60 min. After centrifugation at 1000 rpm for 5 min, the cells were resuspended with 500 μL of 1× PBS and evaluated using flow cytometry (Calibur II, BD Bioscience, USA). The cells were collected using CellQuest software, and the experimental data were analyzed using the Flowing software program. The state of the cells was observed with forward scatter/side scatter, and the fluorescence intensity of the cells was observed with fluorescence channel FL1.

### AO/EB dual fluorescence detection method to detect apoptosis

The AO/EB working solution was prepared, added to each group of cell samples separately, and mixed well. Low-speed centrifugation was used for 5 min, and the supernatant was discarded. The cells were resuspended in AO/EB dilution buffer, the cells counted, and then the cell density was adjusted to 0.5–5 × 10^6^ cells/ml. AO/EB working solution was added to every 25 μl of cell suspension and mixed well. On a clean glass slide, 5 μl of the cell suspension was added dropwise, and then gently covered with glass for observation under a confocal microscope.

### Carboxyfluorescein succinimidyl ester (CFSE) cell proliferation activity detection

The CFSE cell labeling solution was diluted 10 times with pure water. According to the number of cells samples to be tested, the CFSE mother liquor was diluted 100**–**500 times with the cell labeling solution and then diluted 2–8 times with PBS to prepare the staining working solution. Finally, the CFSE mother liquor was diluted 200**–**4000 times. The optimal dyeing concentration (1:500) was determined according to the results of the preliminary experiment. The cells to be tested were resuspended with the staining working solution to a cell concentration of approximately 10^7^/ml and then incubated at 37 °C for 15**–**30 min. After centrifugation, the supernatant was removed to collect the cells. PBS or serum-free medium was added to resuspend the cells, and 500 μl of the cell suspension was taken and used for detection in the flow cytometer.

### Hematoxylin–eosin staining

Hematoxylin and eosin (H&E) staining and Masson staining were performed on the pancreatic tissues of rats in the four groups.

### Bioinformatics analysis

mRNA and LnRNA biochip detection on the AP group and control group were performed to obtain a gene expression matrix. The limma package in Bioconductor was used to analyze differentially expressed genes. A false-discovery rate (FDR) < 0.05 and | log2FC | ≥1 were set. We performed GO enrichment and KEGG functional enrichment analysis of differential genes (*p* < 0.05). Protein–protein interaction (PPI) network mapping of all differential genes was performed by GENEMANIA, and MCC calculation was performed by CytoHub software to screen Hub genes. We obtained miRNA sequencing data (GSE24279) and mRNA data from normal people and AP patients from the GEO database for difference analysis (an FDR of <0.05 and | log_2_ FC | ≥ 0.58 were set). We predicted that the miRNA may bind to Trim33 by starBase and TargetScan. We constructed a co-expression network of differential mRNA and LnRNA. LncBase Predicted v.2 (http://carolina.imis.athena-innovation.gr/diana_tools/web/index.php?r=lncbasev2%2Findex-predicted) predicted that lncRNA TCONS_00021785 had a binding effect with miR-21-5p. The mechanism diagram is drawn by Biorender.

### Statistical analysis

The experimental data are expressed as the mean ± standard deviation (*x* ± *s*). The *t*-test was used to compare the means of two samples.(***p < 0.001, **p < 0.01, *p < 0.05, and *p* < 0.05 meant the difference was statistically significant).

## Supplementary information

Construct a PPI network and look for the core gene Trim33.

## Data Availability

Data are available from the GEO database (GSE24279) and the corresponding author.
