# Supplementary figures and images for: The lncRNA TCONS_00021785/miR-21-5p/Trim33 axis regulates VMP1-mediated zymophagy, reduces the activation of trypsinogen, and promotes acinar cell recovery
Source: Cell Death Discov. 2022 Feb 15;8:65. doi: 10.1038/s41420-022-00862-4 (PMC8847645; doi:10.1038/s41420-022-00862-4)

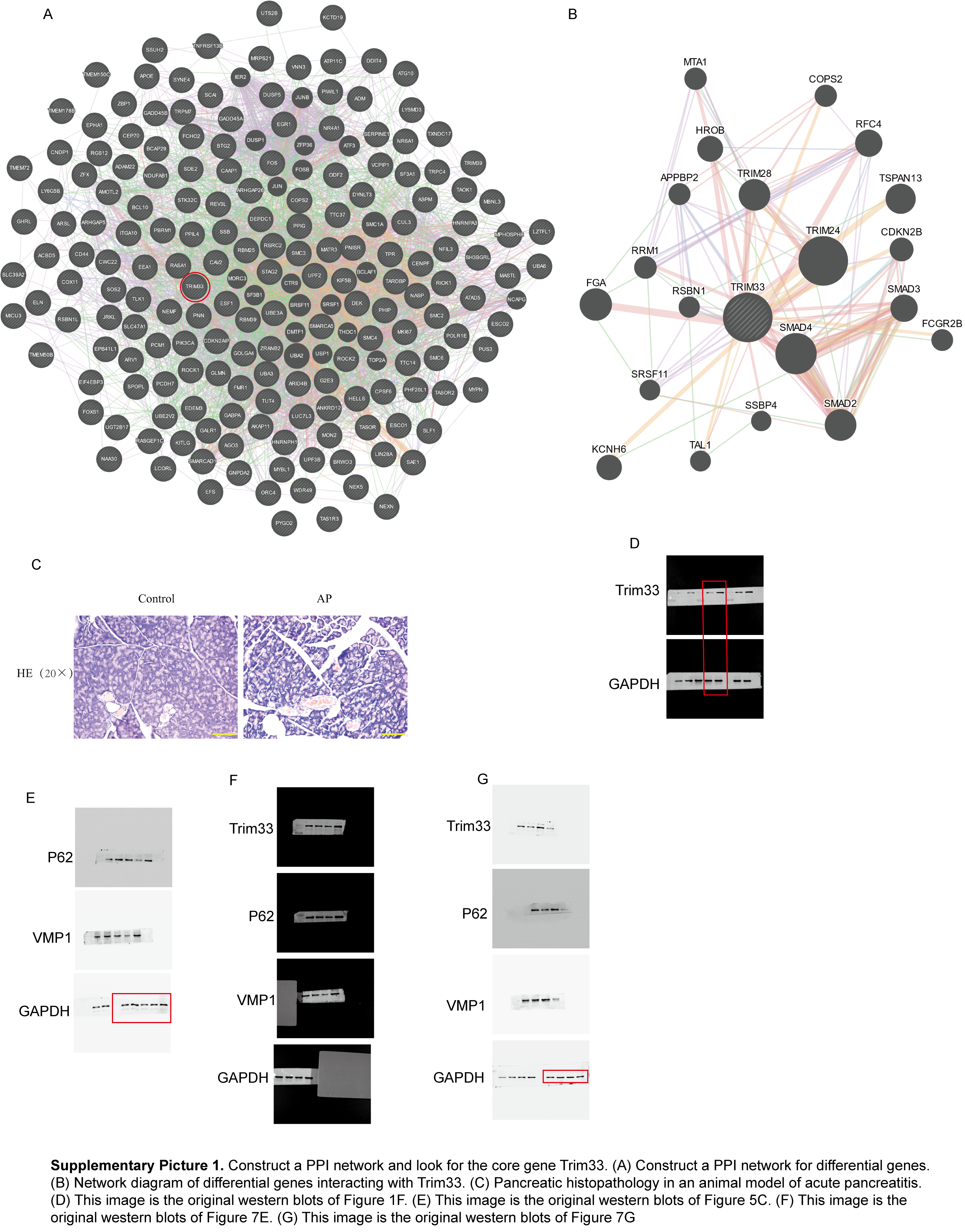

Supplement: Supplementary file 1 — Construct a PPI network and look for the core gene Trim33. [file 41420_2022_862_MOESM1_ESM.tif]
